# A new approach to produce IgG_4_-like bispecific antibodies

**DOI:** 10.1038/s41598-021-97393-2

**Published:** 2021-09-20

**Authors:** Caizhi Zhao, Wei Zhang, Guihua Gong, Liping Xie, Ming-Wei Wang, Youjia Hu

**Affiliations:** 1 School of Pharmacy, Fudan University, Shanghai, 201203 China; 2 China State Institute of Pharmaceutical Industry, Shanghai, 201203 China; 3 The National Center for Drug Screening, Shanghai, 201203 China

**Keywords:** Biotechnology, Immunology

## Abstract

While achieving rapid developments in recent years, bispecific antibodies are still difficult to design and manufacture, due to mispair of both heavy and light chains. Here we report a novel technology to make bispecific molecules. The knob-into-hole method was used to pair two distinct heavy chains as a heterodimer. IgG_4_ S228P CH1-CL interface was then partially replaced by T-cell receptor α/β constant domain to increase the efficiency of cognate heavy and light chain pairing. Following expression and purification, the bispecific antibody interface exchange was confirmed by Western blotting and LC–MS/MS. To ensure its validity, we combined a monovalent bispecific antibody against PD-1 (sequence from Pembrolizumab) and LAG3 (sequence from Relatlimab). The results showed that the molecule could be assembled correctly at a ratio of 95% in cells. In vitro functional assay demonstrated that the purified bispecific antibody exhibits an enhanced agonist activity compared to that of the parental antibodies. Low immunogenicity was predicted by an open-access software and ADA test.

## Introduction

As a key component of monoclonal antibody therapy^1^, bispecific antibodies have been developed rapidly as a new strategy for cancer therapy^2–4^. Monoclonal antibodies are monospecific, can bind to only one antigen and have limited target specificity. However, bispecific antibodies, by design, can bind to two different antigens or two different epitopes of the same antigen to maximize the specificity, thereby greatly expanding therapeutic scope and potential^3^.

It is known that bispecific antibodies are difficult to produce in a single-cell system upon co-expression. Mispairing of heavy and light chains usually leads to low yield^5^. Conventional bispecific antibodies are made by chemical conjugation such as connecting the two monoclonal antibodies together or in the form of a hybridoma^6,7^. Several techniques were applied nowadays to form heterogeneous heavy chains including knob-into-hole^8^, charge interaction at CH3^9^, replacement of CH3 domain^10^. These involve a series of engineering processes such as heterodimerization of heavy and light chains^11^, replacement of CL and CH1 domains^12^, application of linkers to connect heavy and light chains^13^, assembly of heavy and light chains from two monoclonal antibodies in vitro^14,15^ and transformation of Fab domain^16^. For instance, a recently reported bispecific antibody against EGFR and IGFR is a single-domain Fab that connects the light chain to the heavy chain via a 32-amino acid linker^17^. It is of note that this novel method facilitates the combination of a light chain with its cognate heavy chain by substituting CH1 and CL domains of one Fab arm with Cα and Cβ domains of the T-cell receptor (TCR). The rationale behind this approach is that TCR has a similar heterologous structure as Fab, and their constant region structure is also homologous to IgG CH1/CL^18^. TCR has been widely utilized in antibody therapy, for example, the variable domain of a monoclonal antibody was combined with the constant domain of TCR to form recombinant and antibody guided T cells^19–22^. In addition, BEAT technology using the TCR α/β constant domain was applied to solve heterodimers of heavy chains^23^.

In this study, we used knob-into-hole technique to achieve heavy chain heterodimerization through enhancement of heavy and light chain pairing and formation of monovalent IgG-like bispecific antibody by partial replacement of CH1-CL interface with amino acids of TCR α/β constant domain interface. Biochemical, biophysical and functional characterization was carried out to profile a bispecific antibody against both PD-1 and LAG3. To our knowledge, this is the first description of using TCR α/β constant domain interface to overcome heavy and light chain mispairing which may provide a new platform to produce different types of bivalent/bispecific IgGs.

## Results

### Mutation design

**Figure 1:**
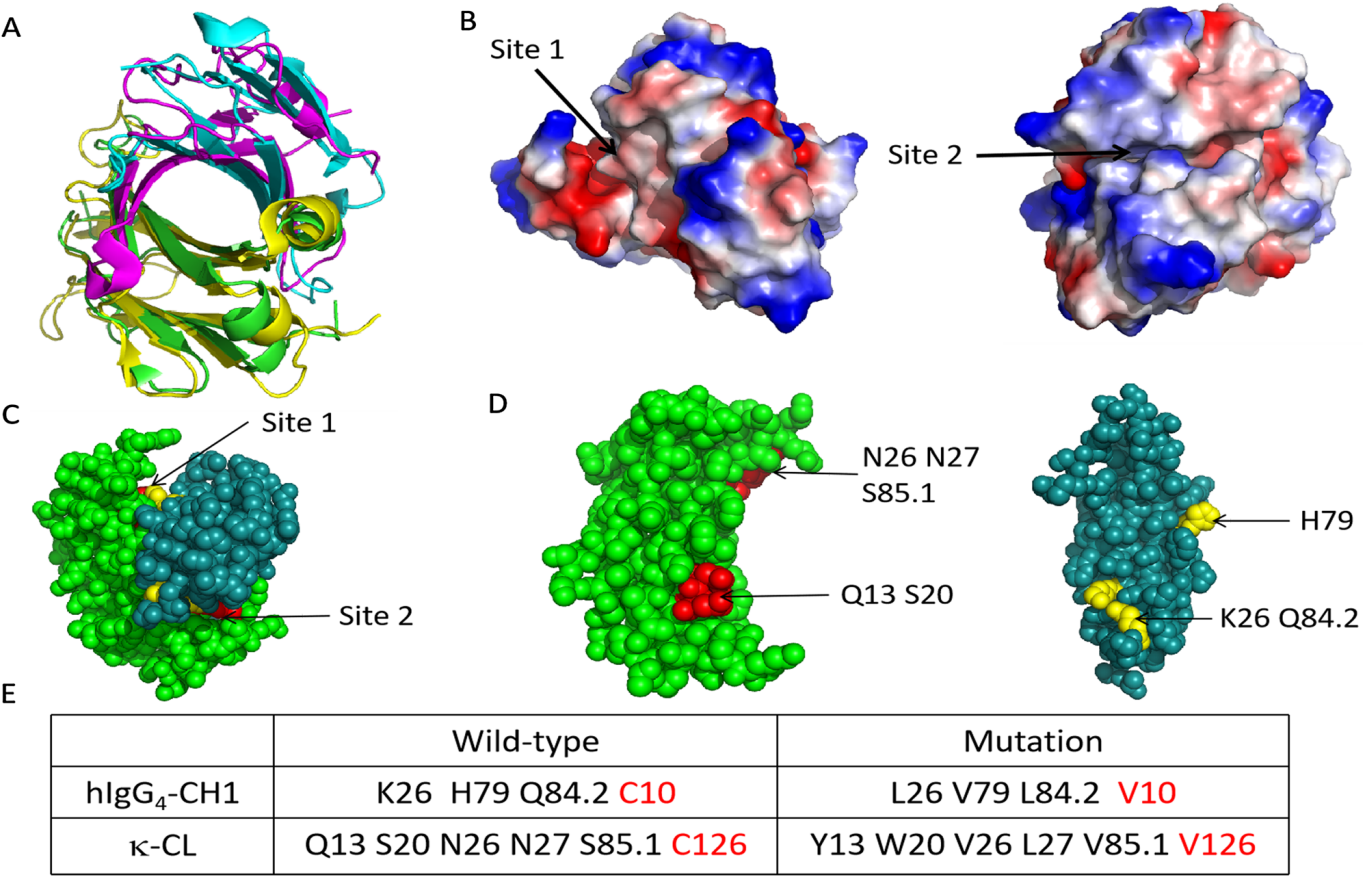
Amino acid analysis and mutations in the CH1-CL interface. (**A**) Structural overlay of CH1/CL with TCR Cα/Cβ. Green and blue represent CL and CH1, respectively. Purple and yellow represent Cα and Cβ, respectively. (**B**) The crystal structure of CH1-CL was analyzed by electrostatic potential. Red represents negatively charged amino acids, blue represents positively charged amino acids, and white represents non-polar amino acids. The color change from red to blue represents the trend of amino acids from negatively charged to positively charged. The color shows that the charge and hydrophobic action of amino acids at Site 1 and Site 2 are weak. (**C**) The spherical structure of Site 1 and Site 2 at both ends of the Interface. Red and yellow represent CL and CH1 mutated amino acids, respectively. (**D**) Specific locations. (**E**) The amino acids of CH1-CL and their positions grafted from TCR. Red represents the position of native disulfide bond and the mutated amino acids.

We selected IgG_4_ S228P as the development target (PDB: 5DK3) and analyzed the overlay crystal structures of CH1/CL and TCR Cα/Cβ (PDB: 3ARB) (Fig. 1A). It was found that there were two sites where the interaction force was relatively weak in the CH1-CL interface. Sites 1 and 2 have hydrophilic amino acids that weaken the interaction between the two chains (Fig. 1B). We thus grafted two major hydrophobic areas of the TCR α/β constant domains (Supplementary Fig. S1) to the two weak interaction sites of CH1-CL, respectively, in order to obtain correct pairs of heavy and light chains, while leaving strong amino acid interaction in the CH1-CL interface unchanged.

It was found that the weak charge interaction of site 1 occurs among Asn26, Asn27, Ser85.1 of CL and His79 of CH1 (Fig. 1C). We used one of the two major hydrophobic regions of TCR α/β constant domain interface, i.e., Val86, Leu7 and Val22 in Cα and Val22 in Cβ^23^ to enhance the weak interaction. Thus, His79 was replaced by Val in CH1 and Asn27, Asn26 and Ser85.1 by Leu, Val and Val in CL, respectively (Fig. 1D). These mutations constructed a new hydrophobic region in the edge of CH1-CL. At the other end of which, the second largest hydrophobic region of TCR was introduced to include residues Trp88 and Tyr79 in Cα as well as Leu24 and Leu84 in TCR^23^. Similarly, Ser20 in CL was mutated to Trp, which has a large side chain, and Gln13 was mutated to Tyr. Within CH1, basic residue Lys26 and polar residue Gln84.2 were both mutated to non-polar amino acid Leu (Fig. 1D). With the addition of small non-polar residues nearby, another new large hydrophobic region was formed. After analyzing the entire modified CH1-CL structure, two large hydrophobic interaction areas of TCR Cα-Cβ were confirmed to present at both ends of the β sheet within CH1-CL interface. These modifications should in theory strengthen the acting force between CH1 and CL. Moreover, side chains of CH1 (Pro82 and Leu84.1) and CL (Ser81 and Glu79) will form respective polar bonds to stabilize the entire structure.

To prevent mispair of heavy and light chains, the native disulfide bond in CH1-CL was also mutated, and the corresponding Cys was substituted by Val^24^. All amino acids of CH1-CL that were subjected to mutation are shown in Fig. 1E.

### Heterodimer verification

**Figure 2:**
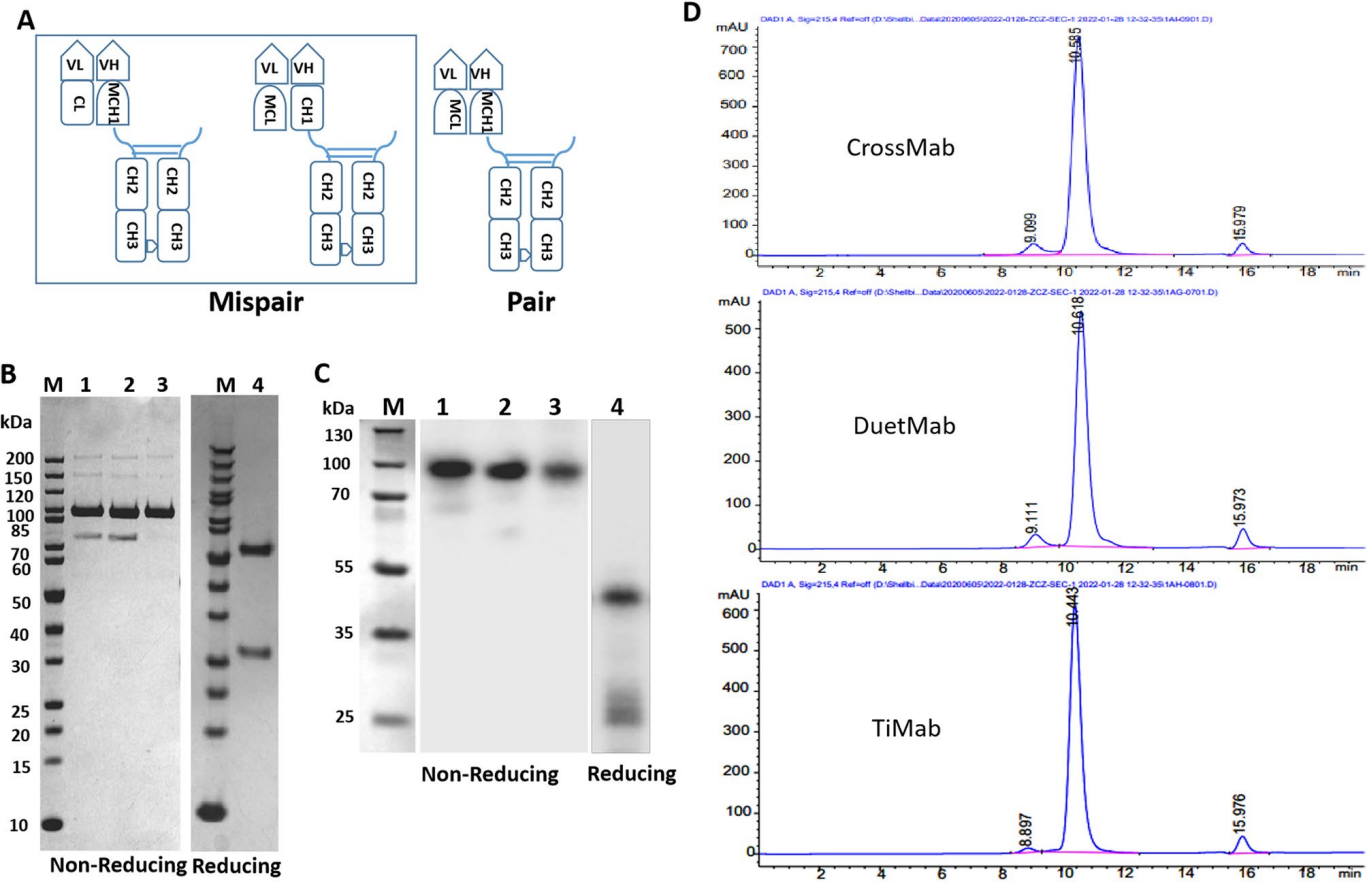
Expression and purification of one value format M. (**A**) The model diagram of M. Mutated CH1 and CL are shown as MCH1 and MCL. Possible mismatch forms are shown. Heterodimerization of distinct heavy chains is achieved by use of the KIH technology. (**B**) 1, 2 and 3 respectively represent the one value M molecules of crossMab, DuteMab and TiMab in our design. The purified SDS-PAGE results show that molecules 1 and 2 could not be completely assembled and the light chain dropped, while molecule 3 was almost completely assembled at an apparent ratio close to 95%. 4 represents the reducing SDS-PAGE of TiMab after purification. The result shows a normal antibody band profile (lane 4). (**C**) Western blots confirmed the identity of the main bands. 1, 2 and 3 respectively represent TiMab, DuetMab and CrossMab. 4 represents the reducing western blot of TiMab. (**D**) SEC-HPLC results showed that the purity of TiMab was higher than CrossMab and DuetMab.

In human IgG-like bispecific antibodies, heavy and light chain mispair is generated because CH1-CL has two arms that form heterodimers with identical sequences when co-expressed in a single cell. To examine if the newly designed molecules could be assembled correctly, we studied all possible mispairs in the molecular format of one value M (Fig. 2A). The constructed plasmids bearing single-strand antibody coding were co-expressed and the results showed that mutated CL and CH1 could not be assembled with that of wild-type (Supplementary Fig. S1), while mutated CH1 and CL were able to assemble correctly (Fig. 2B) with an abundance close to 95%. Western blots confirmed the identity of the main bands (Fig. 2C). The data demonstrate that the heavy chain knob-into-hole was working. The partial heavy chain has the same size as the light chain. The product after protein A purification displayed a relatively single peak and the retention time is consistent with that of the theoretical value (Fig. 2D and Supplementary Fig. S3). We also found that the purity of TiMab was higher than CrossMab and DuetMab (Fig. 2D).

One previous study reported that native disulfide bonds replaced by a pair of non-native disulfide bonds can mitigated the problem of mispair^26^. We repeated this design but found substitution of disulfide bonds did not significantly improve the expression and correct paring of our bispecific antibody (Supplementary Fig. S4).

### Production and validation

Programed death-1 (PD-1) is an inhibitory receptor expressed on the surface of T cells. Inhibitory signal caused by PD-1 binding with its ligands PD-L1 and PD-L2 reduces T cell proliferation, cytokine secretion and cytotoxic activity^25,26^. In multiple syngeneic mouse tumor models, blockade of PD-1 or its ligands promoted the antitumor activity^27–29^, which could be further enhanced by antibodies against other negative regulators of T-cells, such as CTLA-4 and LAG3^30,31^. To produce a bispecific antibody targeting both PD-1 and LAG-3, we selected IgG_4_ S228P^32^ to make the construct.

**Figure 3:**
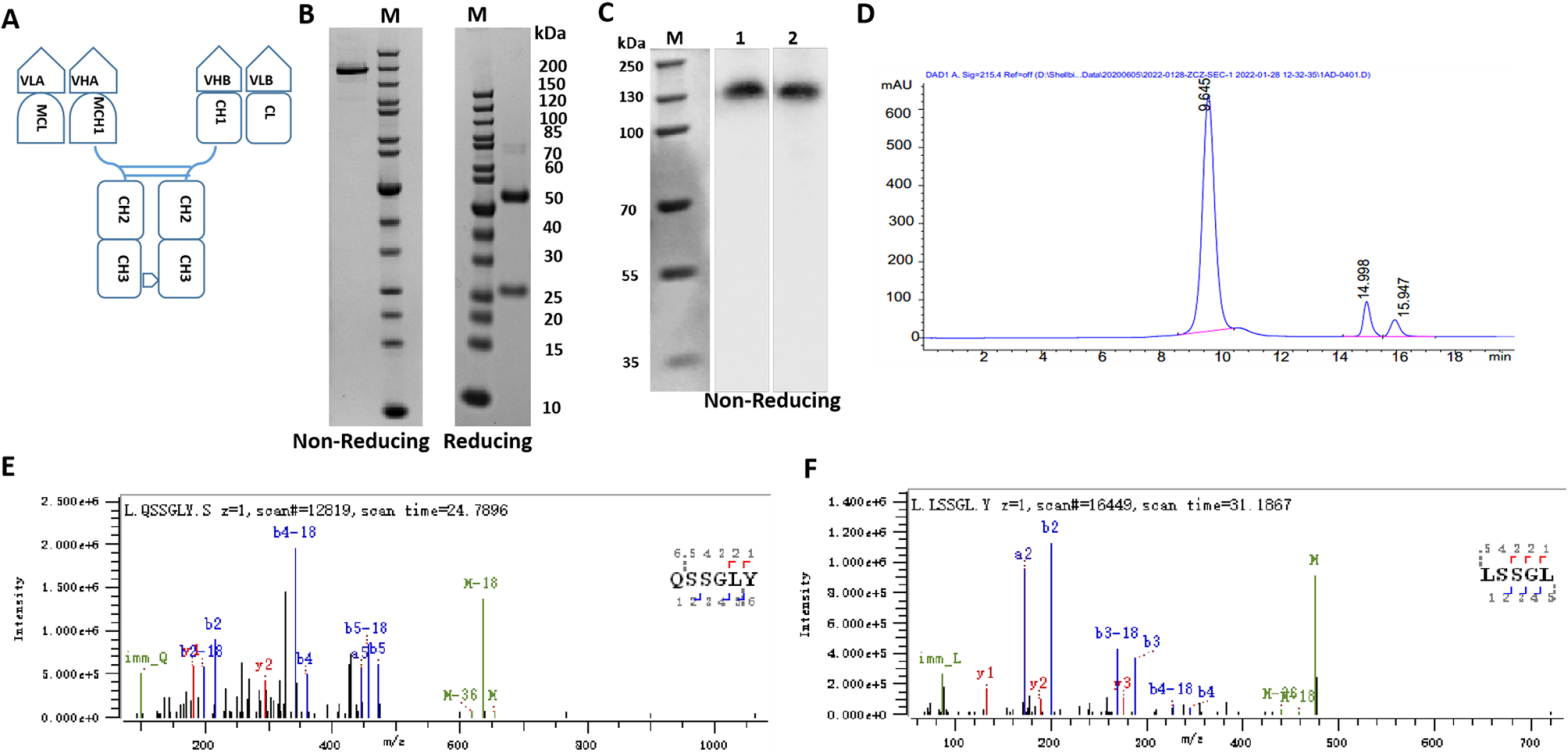
Physicochemical characterization of PD-1 × LAG3 TiMab. (**A**) The molecular form of the bispecific antibody. (**B**) Non-reducing SDS-PAGE of purified protein confirms a purity of 94.5% by gray scanning. The apparent size corresponds to the theoretical molecular weight of 145 kDa. (**C**) Western blot confirms the integrity of the molecule using 1 anti-Fab of LAG3, and 2 anti-Fab of PD-1 as primary antibodies. (**B**) Non-reduced SDS-PAGE confirms the purity of 94.5% by gray scanning. The apparent size corresponds to the theoretical molecular weight of 145 kDa. (**D**) Size-exclusion chromatography of purified antibody shows a single main peak with a purity of 94.2%. Peptide mapping of wild-type CH1. Number 1 represents the first amino acid from the N terminal (**E**) and mutated antibody (**F**) by LC–MS. The amino acid Q was successfully mutated to L.

The PD-1 × LAG3 bispecific antibody (PD-1 × LAG3 TiMab) was produced by transient co-expression of 4 plasmids in Expi293 cells (Fig. 3A). The molar ratio of heavy and light chains was 1:2. Construction of plasmids encoding heavy and light chain genes is described in Materials and Methods. The PD-1 × LAG3 TiMab yield was 30 mg/L (Fig. 3B) while that of PD-1 and LAG3 monospecific antibodies were 120 mg/L and 95 mg/L, respectively. Obviously, the expression level of the bispecific antibody is lower than its parental IgGs. The result of western blotting confirmed the integrity of the molecule and also demonstrated the pairing of heavy chain knob-into-hole (Fig. 3C). PD-1 × LAG3 TiMab was purified using standard protein A affinity chromatography. Sodium dodecyl sulfate polyacrylamide gel electrophoresis (SDS-PAGE) and size exclusion chromatography were employed to analyze the purity and chain composition. Purified PD-1 × LAG3 TiMab showed a single peak with very low levels of aggregates in SEC analysis. Fragments of free chains were also insignificant (Fig. 3D). The peak retention was similar to its parental molecules due to identical molecular mass of IgGs.

We subsequently carried out mass spectrometry to analyze whether the amino acids in the modified CH1-CL of PD-1 × LAG3 TiMab were successfully mutated. Analysis of the different enzyme digested antibody demonstrated that the target residues in CH1 and CL were all mutated according to our design, e.g., Glu (Q) (Fig. 3E) in CH1 was changed to Leu (L) (Fig. 3F; other mutation sites were not shown).

### Binding property

**Figure 4:**
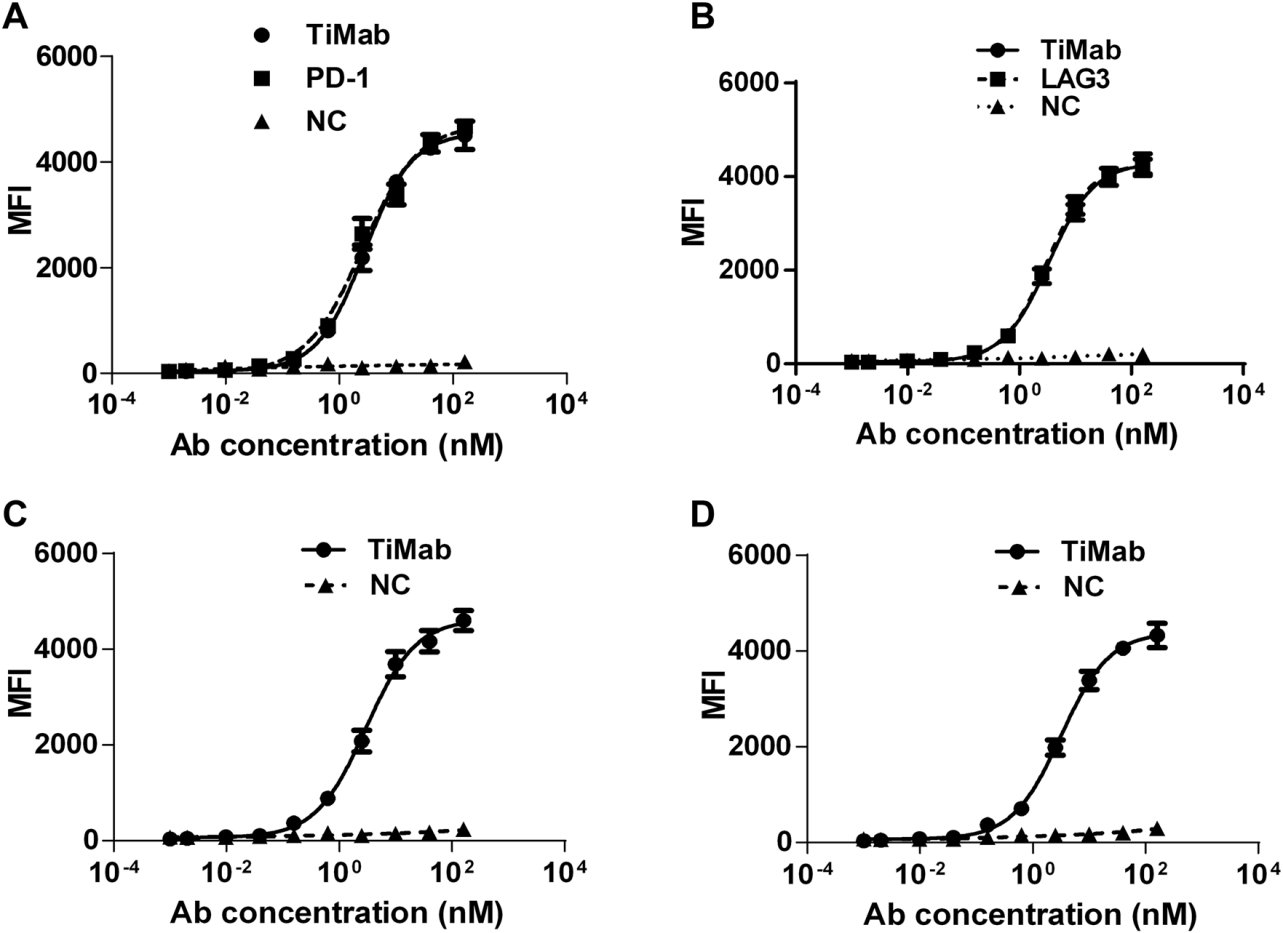
Binding analysis of PD-1 × LAG3 TiMab by FACS. The engineered cells expressing PD-1 (**A**) or LAG3 (**B**) only were incubated separately. Results show that TiMab has a similar antigen-binding ability as that of the parental antibodies. The circle is TiMab, the square is PD-1 × LAG3, and the triangle is negative control (NC). (**C**) PD-1 × LAG3 TiMab molecule was incubated with the engineering cells expressing PD-1 only. LAG3 was used as the detection target. (**D**) PD-1 × LAG3 TiMab molecule was incubated with the engineering cells expressing LAG3 only. PD-1 was used as the detection target. Corresponding antigens were added. The detection results show that the binding level of LAG3 and PD-1 were similar to their parentals, indicating that TiMab is capable of binding to PD-1 and LAG3 simultaneously at the cellular level. Ab, antibody.

Antibody binding affinity to cell surface expressing antigens PD-1 and LAG3 was assessed by flow cytometry (FACS). PD-1 × LAG3 TiMab exhibited similar binding affinity as the parental monospecific antibodies (Fig. 4A,B). It binds to CHO cells expressing PD-1 and LAG3 at 2.76 nM and 3.37 nM, respectively (Supplementary Table 1). The dual binding ability of PD-1 × LAG3 TiMab was demonstrated in engineered cells that can bind to one arm of the antibody while the other arm is open for detection. When PD-1 × LAG3 TiMab was saturated with PD-1 in the engineered cells, addition of LAG3 elicited a second binding signal. By calculating the fluorescence intensity of the binding events, we determined that PD-1 × LAG3 TiMab is capable of binding both antigens simultaneously (Fig. 4C). Furthermore, using PD-1 as detection target also yielded a similar result (Fig. 4D). These data suggest a correct Fab folding in the presence of mutated CH1-CL interface.

We then investigated whether mutations in CH1-CL interface affects Fc-mediated functional activity. Binding affinities of PD-1 × LAG3 TiMab to various human Fcγ receptors and FcRn were determined by a steady-state equilibrium binding assay on ProteOn. As shown in Table 1, the binding kinetics of the antibody to different Fcγ receptors are indistinguishable from the two parental antibodies and an isotype control of human IgG_4_ S228P. The interaction with human FcRn at pH 6 was also shown in Table 1. These data imply that Fc function is maintained following the mutations.

**Table 1:** Equilibrium binding of PD-1 × LAG3 TiMab and parental IgGs to various Fc receptors.

	Antibody KD (nM)			
	FcγRI	FcγRIIa	FcγRIIb	HuFcRn
TiMab	3.8 × 10^–9^	1.1 × 10^–6^	2.3 × 10^–7^	4.2 × 10^–6^
PD-1	3.2 × 10^–9^	9.7 × 10^–7^	2.8 × 10^–7^	5.9 × 10^–6^
LAG3	4.4 × 10^–9^	8.8 × 10^–7^	2.6 × 10^–7^	6.8 × 10^–6^
Isotype	3.7 × 10^–9^	1.4 × 10^–6^	3.1 × 10^–7^	6.2 × 10^–6^

### Thermostability

Thermostability of purified PD-1 × LAG3 TiMab was measured at 1 mg/mL and 2 mL of which were incubated at 40 °C for a specified period. Protein concentration and purity were checked at different time points. Compared to the control antibody, PD-1 × LAG3 TiMab was not degraded significantly (Supplementary Table 2). After incubation at 40 °C for 21 days, it still displayed a similar binding ability as that of the parental (Supplementary Fig. S5A,B). The thermostability was also examined with Uncle biologics stability screening platform (Unchained Labs). As shown in Supplementary Fig. S5C, the melting temperature (Tm1) is 62.37 °C, lower than the control IgG_4_ S228P whose Tm1 is 69.00 °C, indicating that PD-1 × LAG3 TiMab is relatively stable.

### In vitro activity

**Figure 5:**
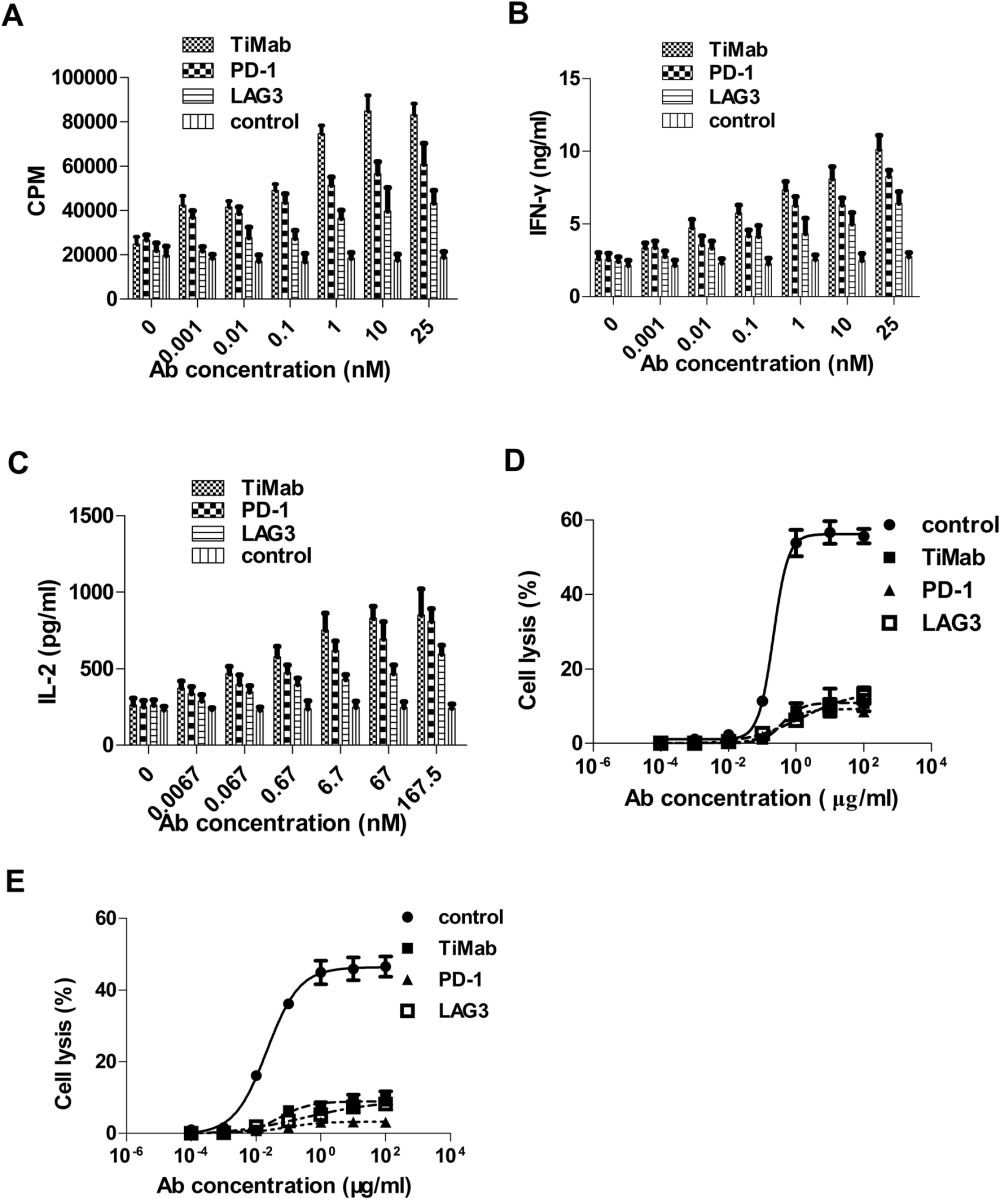
Function assay of PD-1 × LAG-3 TiMab. (**A**) The effects of all the antibodies studied on T-cell proliferation. Activation of the bispecific antibody is higher than that of the parental and is concentration-dependent. IFN-γ (**B**) and IL-2 (**C**) secretion were promoted by the addition of antibodies. ADCC (**D**) and CDC (**E**) assays using activated T cells. Bispecific and parental antibodies had weak ADCC and CDC activities compared with human IgG1 isotype. Data shown are means ± S.D. of multiple measurements. *Ab* antibody.

The bioactivity of PD-1 × LAG3 TiMab was compared to that of the monovalent forms of the parental antibodies. In a human T-cell response assay consisted of an allogeneic MLR, stimulation of human PBMC by super-antigenic DC and antigen-specific stimulation of T cells, blockade of PD-1 and LAG3 by PD-1 × LAG3 TiMab resulted in a titratable enhancement of IFN-γ release (Fig. 5B). In some donor T-cell/DC pairs, enhanced T-cell proliferation was observed (Fig. 5A). It also enhanced IL-2 secretion in response to DC using PBMC compared to the isotype control (Fig. 5C). Taken together, these data demonstrated that PD-1 × LAG3 TiMab can, at a very low concentration, enhance T-cell reactivity in the presence of a TCR stimulus. Specifically, there were significant releases of inflammatory cytokines, including IFN-γ and IL-2, from stimulated PBMC after co-incubation with the antibody.

Additionally, the ability of PD-1 × LAG3 TiMab (0.003–50 mg/mL) to mediate ADCC activity in vitro was tested by using IL-2 activated PBMC as a source of natural killer (NK) cells as well as activated human CD4^+^ T cells expressing high levels of membrane PD-1 and LAG3 as target cells. Compared to a positive control of IgG1 antibody, PD-1 × LAG3 was unable to mediate ADCC of T cells at high concentrations (Fig. 5D). It also failed to mediate complement-mediated cytotoxicity (CDC) of activated human CD4^+^ T cells in the presence of human complement (Fig. 5E).

### Pharmacokinetic profile

**Figure 6:**
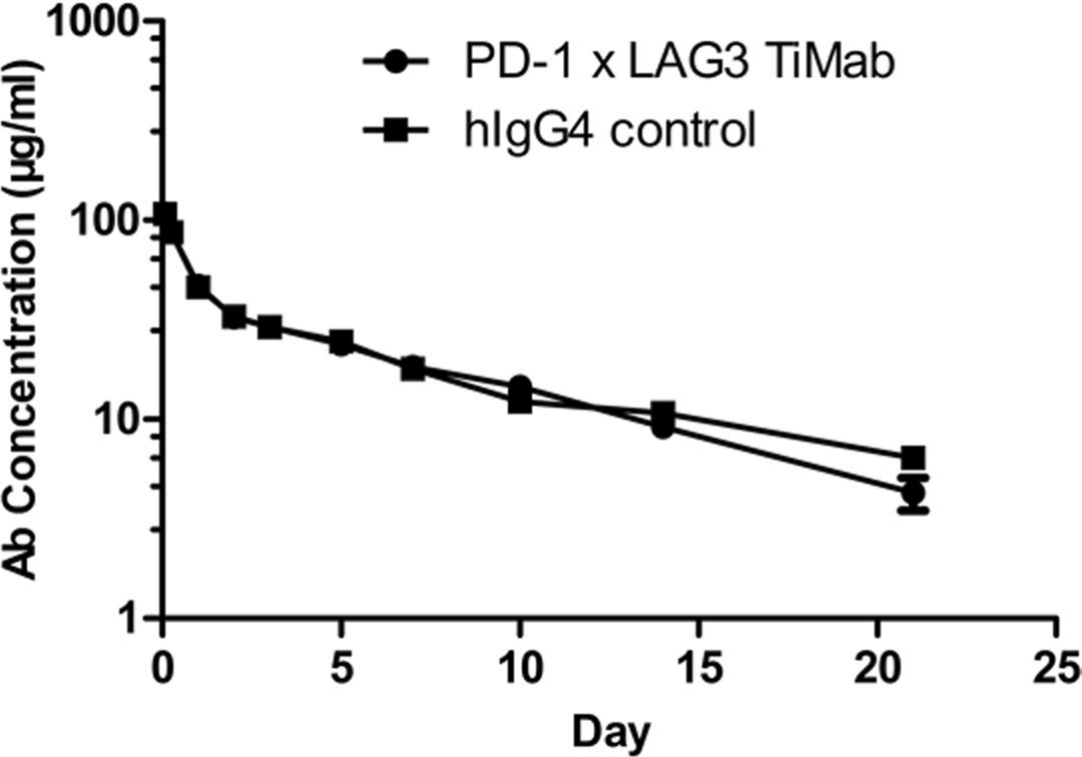
Pharmacokinetics profiles of PD-1 × LAG3 TiMab in SD rats. Pharmacokinetics parameters were determined by non-compartmental analysis using WinNonlin software.

We used a rat model to study the pharmacokinetic properties of PD-1 × LAG3 TiMab by intravenous injection. Its circulating concentrations were determined by measuring that of PD-1 or LAG3 in the animal serum specifically captured by anti-Fc antibodies. In fact that the serum concentration of the bispecific antibody assessed by either antigen was very much alike, indicating that the molecule is intact in vivo and has the ability to bind both antigens. However, it is less stable than conventional IgG at the same intravenous dose (Fig. 6). The drug clearance rate was faster, with a half-life shorter than 10 days. This is expected because of the mutations in the CH1 and CL domains. The long half-life observed is consistent with previous observations on heterodimers formed by knob-into-hole method^33^^.^

### Immunogenicity

**Figure 7:**
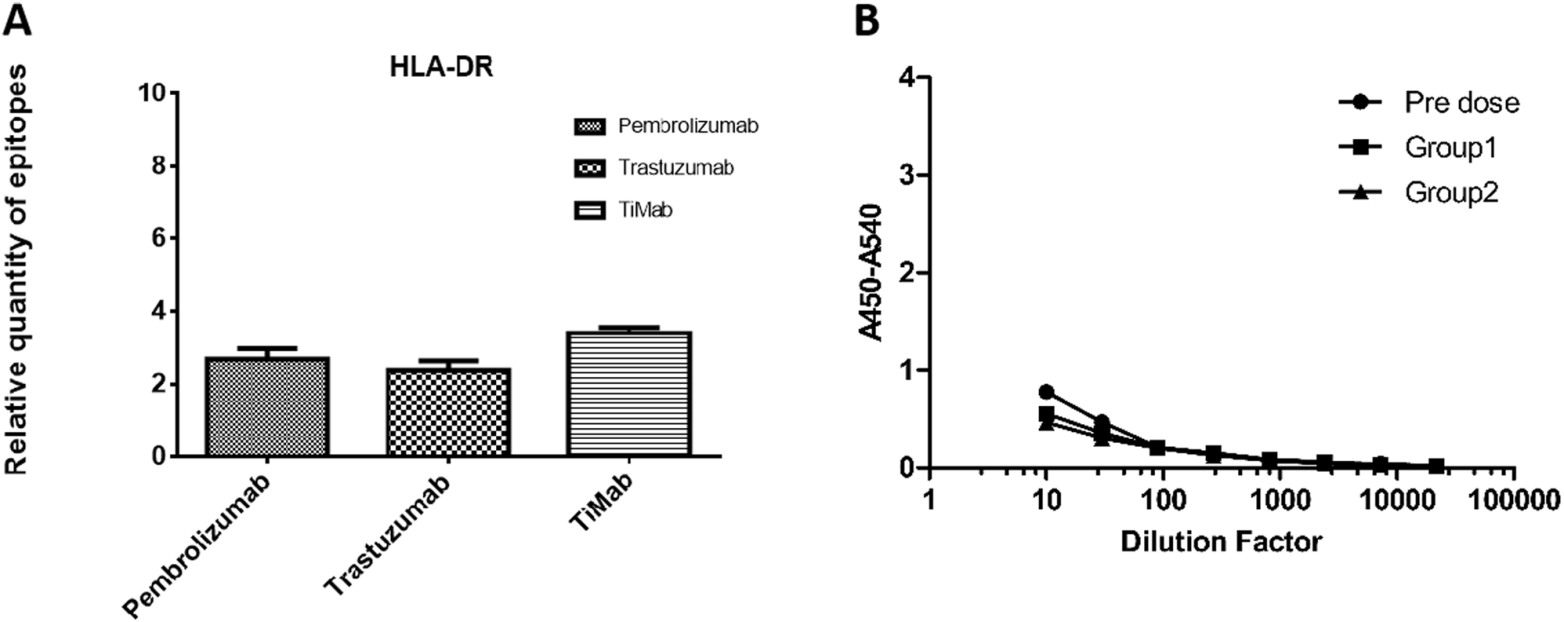
Software prediction and in vivo ADA detection of immunogenicity. (**A**) Software prediction using Pembrolizumab and Trastuzumab as positive control whose immunogenicity profiles are low in the clinic. PD-1 × LAG3 TiMab has a similar low level. (**B**) In vivo detection of ADA. Group 1 and Group 2 produced no obvious ADA. Pre-dose is a negative control without anti-drug antibodies.

We used free online IEDB software to predict the immunogenicity of PD-1 × LAG3 TiMab in comparison with Trastuzumab and Pembrolizumab (Fig. 7A) which are non-immunogenic in the clinic. Immunogenicity can lead to the formation of anti-drug-antibody (ADA) immune complexes thereby affecting drug safety and pharmacokinetics. Therefore, ADA test was performed in PD-1 × LAG3 TiMab coated plates and incubated with rat serum for 14 and 21 days. Mouse-anti-rat IgG was then added to reveal the results showing that our antibody did not produce significant ADA at both time points (Fig. 7B). Combined with the above prediction, introduction of site-directed mutation did not cause observable immunogenicity.

## Discussion

Bispecific antibodies in cancer therapy mainly involve effector cell recruitment such as T cells that could not be accomplished with conventional antibodies^34,35^. Other bispecific antibody based therapeutic strategies may include repositioning effector molecules and cells, attenuating or enhancing ADCC and CDC effects, prolongation of half-life and better penetration of blood–brain barrier^36–40^. These actions are related to the traditional IgG antibody, whose classical structures are of great value in the design of bispecific antibodies.

In this study, we described a new approach to avoid heavy and light chain mispair. This technique is based on analyzing amino acid interaction of CH1-CL and TCR α/β constant domain crystal structures as well as BEAT technology that used the TCR α/β constant domain to solve heterodimers of heavy chains^23^. As shown in Fig. 1B, these two sites are located within the interface, not completely exposed and could be modified without affecting the function. Since TCR α/β constant domains have two major hydrophobic areas (Supplementary Fig. S1), they were introduced to CH3, along with some site mutations, leading to heterodimerization of heavy chains^23^. We partially grafted the existing heterodimer in TCR α/β domains to the CH1-CL while leaving the Fc domain intact. The characteristics of Fc such as long half-life, ADCC, CDC and phagocytosis were thus retained. Notably, several of the mutated residues were not in the CDR region. This design can be applied to IgG_4_ S228P/κ light chains as well as IgG_1_/λ light chains. In addition, it is adaptable to parental sequences from rat, mouse or human without the need of optimization, a significant advantage over scFv and scFab, both contain linker whose length and residue composition require to be optimized.

Although the concept of bispecific antibody has been used for a long time, heavy and light chain mispair is a bottleneck in the development of bispecific antibodies. In the case that heavy chains form heterodimer, the light chains of the two targets of bispecific antibodies are co-transfected into single cells in the instant, such a free combination can produce four forms, of which only one is the correct pair (Supplementary Fig. S6). Therefore, heavy and light chain mispairing is one of the major challenges in obtaining pure bispecific antibodies. The challenge of common light chain is that a large number of filters need to be built, and some targets may be hard to screen to obtain a common light chain^41^. While CrossMab method that exchanges domain VH-VL or CH1-Cl between the heavy and light chain Fab domains can even damage antigen binding ability^42^. In our design, with the application of knob-into-hole to increase the formation of heavy chain heterodimers, two possible mispairs are expressed separately allowing mutated CH1 and CL to assemble in a single cell. Co-expression of three plasmids led to a unique product (Fig. 2) that can be made simply by transient transfection of suspension cells. The purity was close to 95% (Fig. 2D), higher than CrossMab (85%) and DuetMab (80%; Fig. 2D). Sequences of the protein chains can be derived directly from two parental antibodies without the need of further optimization. Because relatively complete Fab is retained, the binding data showed that the affinity of PD-1 × LAG3 TiMab we designed is similar to that of the parental antibodies.

There are different ways to pair heavy and light chains, among which, scFv is most common. Its low molecular weight makes scFv easy to penetrate into various tissues. However, it is relatively unstable and prone to aggregate^43^^.^ The affinity and specificity of scFv are generally lower than that of IgG, and linkers between them may further weaken the activity of VL and VH. Like other single-domain antibodies, scFv may be immunogenic due to the introduction of exogenous linkers^44^. Our design partially grafted the interface of human TCR Cα/Cβ onto CH1-CL. Protein expression and purification did not show any signs of polymerization and aggregation. The protein is thermostable at 40 °C. Supported by ADA assessment, the immunogenicity prediction results are comparable to those drugs already on the market (Fig. 7).

Engineering disulfide bond is a common method to stabilize two interacting chains. Recently, one team analyzed the crystal structure of CH1-CL and found that a pair of newly formed disulfide bonds other than that of the native is important for the interaction between CH1 and CL^26^. Our experimental results demonstrated that by grafting the interface of TCR Cα/Cβ, CH1 and CL can bind well without the dependence on engineered disulfide bonds (Supplementary Fig. S4). It is noteworthy that our design only introduced mutations in CH1 and CL.

In summary, we have developed a new method to generate asymmetric bivalent bispecific IgG-like antibodies. The classical structure is not only simple in purification, but also retains the biological function of Fc. No linker was introduced thereby reducing potential immunogenicity. In addition, this technique also allows the production of symmetric molecules. A tetrevalent (2 + 2) IgG-like bispecific antibody was made by adding wild-type Fab, mutating it to N-terminal or C-terminal of heavy chain (Supplementary Fig. S7) and connecting them via a linker. Co-expression of an extended heavy chain and two light chains are required. The mutation at Fab should enable each light chain to bind to its correct heavy chain partner. This design has the potential to enhance the molecular force in bispecific antibody constructs and is applicable to different molecules.

## Materials and methods

### Animals and ethics statement

Male SD (Sprague–Dawley) rats used in this study were purchased from GemPharmatech (Nanjing, China) and were housed in specific-pathogen free (SPF) barrier facilities which is AAALAC accredited and Office of Laboratory Animal Welfare (OLAW) assured. All animal protocols were approved by the Ethical Committee for Animal Care and Use of China State Institute of Pharmaceutical Industry. All methods are reported in accordance with ARRIVE guidelines. All experiments were carried out according to the Guide for the Care and Use of laboratory animals (National Institutes of Health, USA).

### Construct

We selected IgG_4_ S228P subtype to construct the bispecific antibody (PDB: 5DK3). The amino acid numbers at the interface of Cα/Cβ and CH1-CL refer to IMGT. The sequences of anti-PD-1 and LAG3 were obtained from the patents [WO2015176033A1 and CN105793287A], and the corresponding VL and VH were synthesized by GENEWIZ (Suzhou, China). In order to verify whether our design can correctly assemble the heavy and light chains, we constructed a monovalent IgG and introduced knob-into-hole to solve the heavy chain mismatch and added a stabilizing disulfide bridge in CH3^34^. The sequence of anti-LAG3 was used to construct the monovalent IgG. Mutations in the CH1 and CL domains of H chain (pcDNA3.4-HC.knob) and L chain (pcDNA3.4-LC) transfer vectors were introduced by PCR. Another heavy chain Fc that only has the hinge, CH2 and CH3 was constructed as the hole (pcDNA3.4-Fc.hole)^35^. A total of three molecules were constructed. Non-mutated light chain, heavy chain and Fc were assembled as a positive control molecule; non-mutated light chain, mutated heavy chain and Fc were assembled as a negative control molecule; and mutated light chain, heavy chain and Fc were assembled as a target molecule (Fig. 1).

We also used pcDNA3.4 to construct the monovalent bispecific antibody TiMab against PD-1 and LAG3. The HC of the anti-LAG3 antibody has the knob mutations and that of the anti-PD-1 antibody has the hole mutations in the CH3. The cysteines in the CH1 and CL of the anti-LAG3 antibody were mutated to valines and new mutations were introduced according to the positions shown in Fig. 1E. The CH1 and CL of the anti-PD-1 antibody have wild-type sequences. To determine correct pairing of cognate heavy and light chains, plasmids encoding both antibodies were co-expressed and evaluated for dual specificity by concurrent binding to PD-1 and LAG3 using an FACS assay (described below).

### Expression and purification

Antibodies were produced by transient co-expression of Expi293 cells with pcDNA3.4-Heavy and pcDNA3.4-Light expression vectors (Life Technologies, Carlsbad, USA) in serum-free expression medium (Life Technologies) according to the supplier’s recommended protocol. For co-transfections, plasmids were transfected in the same mass ratio into mammalian cells which were supplemented with enhancer the next day. Cell culture supernatants were harvested 5 days thereafter. SDS-PAGE was used to detect the protein bands. Antibodies were purified by protein A column (GE Healthcare, Pittsburgh, USA) and size exclusion column (GE Healthcare) following buffer exchange in PBS (pH 7.2). Antibody concentration was measured by NanoDrop at 280 nm. The protein purity was analyzed by SDS-PAGE and SEC-HPLC.

#### Western blotting

Western blots were analyzed with goat anti-human Fc-HRP, biotinylated anti-human PD-1 Fab and anti-human LAG3 Fab. The dilution factor of goat anti-human Fc-HRP is 1:10,000 (Abcam). The membranes were cut prior to hybridisation with antibodies.

### LC–MS/MS

Protein samples were digested with different enzymes. The products were desalted using a self-priming desalting column, and the solvent was evaporated in a vacuum centrifuge at 45 °C. The peptides were then dissolved, centrifuged at 13,200 rpm for 10 min at 4 °C, and the supernatant was transferred to the sample tube for mass spectrometry analysis. The raw MS files were analyzed and blasted against target protein database based on the species of the samples using Byonic.

### Antibody stability

Antibodies were incubated at 40 °C on an Eppendorf constant temperature mixer. After 1, 7, 14, and 21 days, the absorption value of protein solution was measured at 280 nm by NanoDrop 2000, the appearance recorded, and the purity detected by SEC-HPLC.

Tm of antibodies was investigated using Uncle Multifunctional protein analyzer (Unchained Labs, USA). The antibody solution (10 μL) was prepared according to the manual and transferred to a 12-well cuvette. The sample was heated from 20 °C to 90 °C at a constant rate of 1 °C/min and the resulting fluorescence data were collected. Tm of the sample was calculated as the average melting temperature of two duplicate wells.

### Antigen binding

Cells with a high expression level of anti-PD-1/LAG3 were cultured and their number was adjusted to 1 × 10^6^ cell/mL before loading to FACS plates (100 μL/well). The bispecific antibody was diluted with 1% BSA for 20 nM and incubated at 100 μL/well. The LAG3/PD-1 antigen with His label was added at the same volume, and the plate was washed after 1 h incubation at 4 °C. The first added antibody was against His and biotin-labeled. The second antibody was then added to read the fluorescence intensity. Both antigens have to be simultaneously bound to the bispecific antibody to give the fluorescence reading thereby verifying its integrity.

### Human cells preparation

Human PBMC and serum were purchased from AllCells (Shanghai, China). Human CD4^+^ T cells were purified from PBMC by negative selection with CD4^+^ T cell enrichment cocktail kit (Stemcell, Vancouver, Canada) according to the manufacturer’s instruction. Human monocytes were isolated from human PBMC by CD14 MicroBeads kit (Miltenyi Biotec, Germany). Immature DC (iDC) was generated from monocytes by culturing with GM-CSF and IL-4 for 5 days and mature DC (mDC) was differentiated by stimulation with LPS at 1 μg/mL overnight. Human activated T cells were separated from human PBMC by T cells Activation kit (Miltenyi Biotec, Germany).

### T-cell proliferation

The effects of PD-1 × LAG3 TiMab on T-cell proliferation was tested by an allogeneic (Miltenyi Biotec, Germany). Primary dendritic cell (DC)-stimulated MLR was conducted in 96-well U-bottom tissue culture plates. Each well has 200 μL RPMI 1640 containing 10% FBS and antibiotics. DCs were mixed with 1 × 10^5^ allogeneic CD4^+^ T cells at a ratio between 1:10 and 1:100. Cells were cultured in the presence or absence of neutralizing PD-1 × LAG3 TiMab and control antibodies (10 μg/mL). The plates were incubated for 5 days, and 16 h before the end of the culturing [^3^H]thymidine was added (1 μCi/well). [^3^H]thymidine incorporation was measured by scintillation counting and T cell proliferation was expressed as the mean [^3^H]thymidine incorporation (counts per minute, CPM) of triplicate wells. Counts due to DCs proliferation alone were routinely lower than 1000 cpm^32,45,46^.

### Cytokine secretion

Human CD4^+^ T cells were mixed with iDC/mDC at a ratio between 10:1 and 100:1. Cells were cultured in the presence or absence of PD-1 × LAG3 TiMab and control antibodies. After 5 days, the supernatants from each culture were harvested for IFN-γ or IL-2 measurement by ELISA. Maxisorp plates were coated with anti-human IFN-γ or IL-2 monoclonal antibody diluted in coating buffer (0.75 μg/mL) to 50 μL/well (i.e., for a 96-well plate adding 3.7 μL of antibody to 5 mL of coating buffer) and incubated overnight at 4 °C. Spare protein binding capacity was blocked by adding 200 μL/well of blocking buffer for 2 h. Dilutions of IFN-γ or IL-2 were used as standards. Two-fold serial dilutions were made from 8000 pg/mL down to 125 pg/mL in complete medium. After washing, standards and test supernatants (100 μL/well) were added and incubated for 2–3 h. The biotinylated anti-IFN-γ or IL-2 monoclonal antibody (1:1333) in blocking buffer was introduced followed by the addition of the extra-avidin peroxidase. The reaction was developed by adding TMB substrate and stopped with 2 M HCl. Absorbance was measured at 450 nm.

### ADCC

Activated T cells were used as target cells and incubated with various concentrations of human antibodies in 96-well plates for 30 min. Then PBMCs as a source of NK cells were added at the ratio of 50:1. The plates were incubated for 6 h at 37 °C in a 5% CO_2_ incubator. Target cell lysis was determined by cytotoxicity detection kit (Roche, Mannheim, Germany). Optical density was measured by a SpectraMax M5e plate reader (Molecular Devices, Sunnyvale, USA).

### CDC

Activated T cells, diluted human serum (AllCells, China) complement and various concentrations of human antibodies were mixed in a 96-well plate for incubation for 4 h at 37 °C in a 5% CO_2_ incubator. Target cell lysis was determined by Cell Titer glo (Promega, Madison, USA).

### ADA

ADA was measured by ELISA. Target proteins (1 μg/mL) were coated and diluted rat serum was added at different time points. Mouse-anti-rat-IgG-HRP was then introduced followed by absorbance reading. OD ratio (S/N) < 2: no ADA; OD ratio ≥ 2: ADA.

### Pharmacokinetics

The pharmacokinetic properties of PD-1 × LAG3 TiMab were analyzed in male SD rats. A total of 12 rats were divided into two groups. After a single dose (10 mg/kg) of intravenous injection, serum samples were taken at different time points and the antibody concentration in the rat serum was detected by ELISA. Goat anti-human Fab was used to capture the target antibody and biotin-conjugated mouse anti-human IgG Fc (Sigma, St. Louis, USA) was used for detection. Plates were read on an EnVision plate reader (PerkinElmer, Boston, USA) and the pharmacokinetic parameters were analyzed by non-compartmental model using WinNonlin software. The results were represented by the mean plus SD.

## Supplementary Information

Supplementary Information.

## References

[1] Sliwkowski MX, Mellman I (2013). Antibody therapeutics in cancer. Science.

[2] Garber K (2014). Bispecific antibodies rise again. Nat. Rev. Drug Discov..

[3] Runcie K, Budman DR, John V, Seetharamu N (2018). Bi-specific and tri-specific antibodies—The next big thing in solid tumor therapeutics. Mol. Med..

[4] Sergey S, Victor P, Valentina B, Georgy N (2018). Bispecific antibodies: Design, therapy, perspectives. Drug Des. Dev. Ther..

[5] Jackman J (2010). Development of a two-part strategy to identify a therapeutic human bispecific antibody that inhibits IgE receptor signaling. J. Biol. Chem..

[6] Brennan M, Davison PF, Paulus H (1985). Preparation of bispecific antibodies by chemical recombination of monoclonal immunoglobulin G1 fragments. Science.

[7] Milstein C, Cuello AC (1983). Hybrid hybridomas and their use in immunohistochemistry. Nature.

[8] Ridgway JB, Presta LG, Carter P (1996). 'Knobs-into-holes' engineering of antibody CH3 domains for heavy chain heterodimerization. Protein Eng..

[9] Gunasekaran K (2010). Enhancing antibody Fc heterodimer formation through electrostatic steering effects: Applications to bispecific molecules and monovalent IgG. J. Biol. Chem..

[10] Davis JH (2010). SEEDbodies: Fusion proteins based on strand-exchange engineered domain (SEED) CH3 heterodimers in an Fc analogue platform for asymmetric binders or immunofusions and bispecific antibodies. Protein Eng. Des. Sel..

[11] Merchant AM (1998). An efficient route to human bispecific IgG. Nat. Biotechnol..

[12] Schaefer W (2011). Immunoglobulin domain crossover as a generic approach for the production of bispecific IgG antibodies. Proc. Natl. Acad. Sci. U. S. A..

[13] Wranik BJ (2012). LUZ-Y, a novel platform for the mammalian cell production of full-length IgG-bispecific antibodies. J. Biol. Chem..

[14] Spiess C (2013). Bispecific antibodies with natural architecture produced by co-culture of bacteria expressing two distinct half-antibodies. Nat. Biotechnol..

[15] Strop P (2012). Generating bispecific human IgG1 and IgG2 antibodies from any antibody pair. J. Mol. Biol..

[16] Lewis SM (2014). Generation of bispecific IgG antibodies by structure-based design of an orthogonal Fab interface. Nat. Biotechnol..

[17] Schanzer JM (2014). A novel glycoengineered bispecific antibody format for targeted inhibition of epidermal growth factor receptor (EGFR) and insulin-like growth factor receptor type I (IGF-1R) demonstrating unique molecular properties. J. Biol. Chem..

[18] Wu X (2015). Protein design of IgG/TCR chimeras for the co-expression of Fab-like moieties within bispecific antibodies. MAbs.

[19] Brocker T, Karjalainen K (1998). Adoptive tumor immunity mediated by lymphocytes bearing modified antigen-specific receptors. Adv. Immunol..

[20] Gross G, Waks T, Eshhar Z (1989). Expression of immunoglobulin-T-cell receptor chimeric molecules as functional receptors with antibody-type specificity. Proc. Natl. Acad. Sci. U. S. A..

[21] Kuwana Y (1987). Expression of chimeric receptor composed of immunoglobulin-derived V regions and T-cell receptor-derived C regions. Biochem. Biophys. Res. Commun..

[22] Porter DL, Levine BL, Kalos M, Bagg A, June CH (2011). Chimeric antigen receptor-modified T cells in chronic lymphoid leukemia. N. Engl. J. Med..

[23] Skegro D (2017). Immunoglobulin domain interface exchange as a platform technology for the generation of Fc heterodimers and bispecific antibodies. J. Biol. Chem..

[24] Mazor Y (2015). Improving target cell specificity using a novel monovalent bispecific IgG design. MAbs.

[25] Brown JA (2003). Blockade of programmed death-1 ligands on dendritic cells enhances T cell activation and cytokine production. J. Immunol..

[26] Freeman GJ (2000). Engagement of the PD-1 immunoinhibitory receptor by a novel B7 family member leads to negative regulation of lymphocyte activation. J. Exp. Med..

[27] Curiel TJ (2003). Blockade of B7–H1 improves myeloid dendritic cell-mediated antitumor immunity. Nat. Med..

[28] Hirano F (2005). Blockade of B7–H1 and PD-1 by monoclonal antibodies potentiates cancer therapeutic immunity. Cancer Res..

[29] Nomi T (2007). Clinical significance and therapeutic potential of the programmed death-1 ligand/programmed death-1 pathway in human pancreatic cancer. Clin. Cancer Res..

[30] Curran MA, Montalvo W, Yagita H, Allison JP (2010). PD-1 and CTLA-4 combination blockade expands infiltrating T cells and reduces regulatory T and myeloid cells within B16 melanoma tumors. Proc. Natl. Acad. Sci. U. S. A..

[31] Woo SR (2012). Immune inhibitory molecules LAG-3 and PD-1 synergistically regulate T-cell function to promote tumoral immune escape. Cancer Res.

[32] Wang C (2014). In vitro characterization of the anti-PD-1 antibody nivolumab, BMS-936558, and in vivo toxicology in non-human primates. Cancer Immunol. Res..

[33] Xu Y (2015). Production of bispecific antibodies in "knobs-into-holes" using a cell-free expression system. MAbs.

[34] de Gast GC, van de Winkel JG, Bast BE (1997). Clinical perspectives of bispecific antibodies in cancer. Cancer Immunol. Immunother..

[35] van Spriel AB, van Ojik HH, van De Winkel JG (2000). Immunotherapeutic perspective for bispecific antibodies. Immunol. Today.

[36] Kontermann RE (2011). Strategies for extended serum half-life of protein therapeutics. Curr. Opin. Biotechnol..

[37] Kontermann RE (2012). Dual targeting strategies with bispecific antibodies. MAbs.

[38] Lameris R (2014). Bispecific antibody platforms for cancer immunotherapy. Crit. Rev. Oncol. Hematol..

[39] Niewoehner J (2014). Increased brain penetration and potency of a therapeutic antibody using a monovalent molecular shuttle. Neuron.

[40] Pardridge WM (2016). Re-engineering therapeutic antibodies for Alzheimer's disease as blood–brain barrier penetrating bi-specific antibodies. Expert Opin. Biol. Ther..

[41] Atwell S, Ridgway JB, Wells JA, Carter P (1997). Stable heterodimers from remodeling the domain interface of a homodimer using a phage display library. J. Mol. Biol..

[42] Chailyan A, Marcatili P, Tramontano A (2011). The association of heavy and light chain variable domains in antibodies: Implications for antigen specificity. FEBS J.

[43] Orcutt KD (2010). A modular IgG-scFv bispecific antibody topology. Protein Eng. Des. Sel..

[44] Vaneycken I (2010). In vitro analysis and in vivo tumor targeting of a humanized, grafted nanobody in mice using pinhole SPECT/micro-CT. J. Nucl. Med..

[45] Chiriva-Internati M (2010). Cancer testis antigen vaccination affords long-term protection in a murine model of ovarian cancer. PLoS One.

[46] Zheng Y (2004). CD86 and CD80 differentially modulate the suppressive function of human regulatory T cells. J. Immunol..

