# Author Correction: A new approach to produce IgG_4_-like bispecific antibodies

**DOI:** 10.1038/s41598-022-07339-5

**Published:** 2022-03-01

**Authors:** Caizhi Zhao, Wei Zhang, Guihua Gong, Liping Xie, Ming‑Wei Wang, Youjia Hu

**Affiliations:** 1 School of Pharmacy, Fudan University, Shanghai, 201203 China; 2 China State Institute of Pharmaceutical Industry, Shanghai, 201203 China; 3 The National Center for Drug Screening, Shanghai, 201203 China

Correction to: *Scientific Reports* 10.1038/s41598-021-97393-2, published online 20 September 2021

The original version of this Article contained errors.

As a result of miscommunication between the Authors, the Authors did not secure permission for the publication of Figures S3, which showed standards and the control antibody in SEC-HPLC, and S5C, which confirmed thermostability of the tested antibody. The experiments shown in these figures were re-ran and the article updated as follows.

In the Results, subheading ‘Thermostability',

"The thermostability was also examined with differential scanning fluorimetry (DSF). As shown in Supplementary Fig. S5C, the melting temperature (Tm1) is 62.45 °C, lower than the control IgG_4_ S228P whose Tm1 is 69.00 °C, indicating that PD-1 × LAG3 TiMab is relatively stable."

now reads:

"The thermostability was also examined with Uncle biologics stability screening platform (Unchained Labs). As shown in Supplementary Fig. S5C, the melting temperature (Tm1) is 62.37°C, lower than the control IgG_4_ S228P whose Tm1 is 69.00°C, indicating that PD-1 × LAG3 TiMab is relatively stable."

In Materials and Methods, under subheading ‘Antibody stability',

"Tm of antibodies was investigated using QuantStudio™ 7 Flex real-time PCR system (Applied Biosystems, Carlsbad, USA). The antibody solution (19 μL) was mixed with 1 μL of 62.5 × SYPRO orange solution (Invitrogen, Carlsbad, USA) and transferred to a 96-well plate (Applied Biosystems). The plate was heated from 26 to 95 °C at a constant rate of 0.9 °C/min and the resulting fluorescence data were collected. The negative derivatives of the fluorescence changes with respect to different temperatures were calculated and the maximal value was defined as melting temperature (Tm)."

now reads:

"Tm of antibodies was investigated using Uncle Multifunctional protein analyzer (Unchained Labs, USA). The antibody solution (10 μL) was prepared according to the manual and transferred to a 12-well cuvette. The sample was heated from 20°C to 90°C at a constant rate of 1°C/min and the resulting fluorescence data were collected. Tm of the sample was calculated as the average melting temperature of two duplicate wells."

Figure S3 and S5C were replaced with the results of replicate runs. In the legend of Figure S5,

"C, The Tm1 of protein was quantitatively detected by DSF. The red arrow indicates a Tm1 value of 62.45°C."

now reads:

"C, The Tm1 of protein was quantitatively detected by Uncle biologics stability screening platform. The red arrow indicates an average Tm1 value of 62.37°C."

Additionally, since Figure 2D and Figure 3D were derived from the same measurement as Figure S3, these figures were also updated with the new measurements.

The conclusions of the Article are unaffected by these changes.

The original version of the Article and accompanying Supplementary Information file have been corrected.

