# Supplementary material for: A new approach to produce IgG4-like bispecific antibodies
Source: Sci Rep. 2021 Sep 20;11:18630. doi: 10.1038/s41598-021-97393-2 (PMC8452627; doi:10.1038/s41598-021-97393-2)

**Supplementary materials**


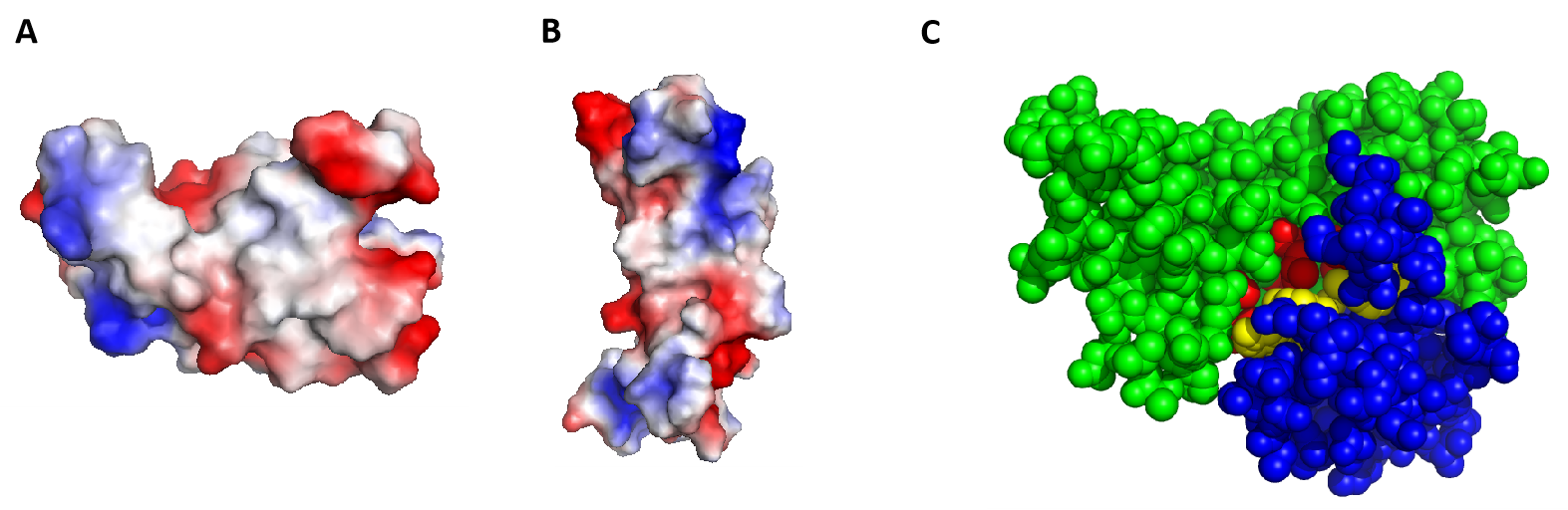


**Supplement Figure 1. The crystal structure of TCR α/β constant domain (PDB: 3ARB).** A and B are electrostatic potential of α constant domain and β constant domain, respectively. C is a sphere showing two big hydrophobic domains in the interface of TCR α/β constant domain. Red and yellow are the hydrophobic amino acids of β and α constant domain, respectively.


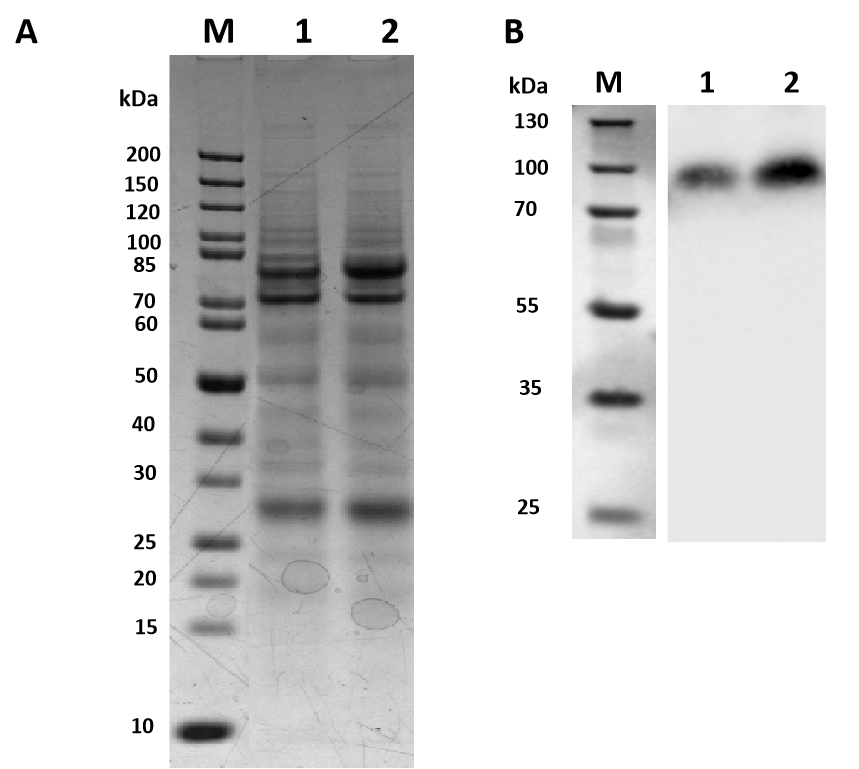


**Supplement Figure 2. Possible pairing expression.** A, SDS-PAGE confirms that mutated CH1 could not pair with wild-type CL and CL could not pair with wild-type CH1 as well. Lanes 1 and 2 are two different possible pairs. Wild type CL could form trace amount of dimer at 50 kd, and mutated CL had almost nothing. B, Western blot demstrated the possible pairs at 75 kd, and have no light chains. 1 and 2 were purified protein materials and anti-human Fc was used as the primary antibody.

**
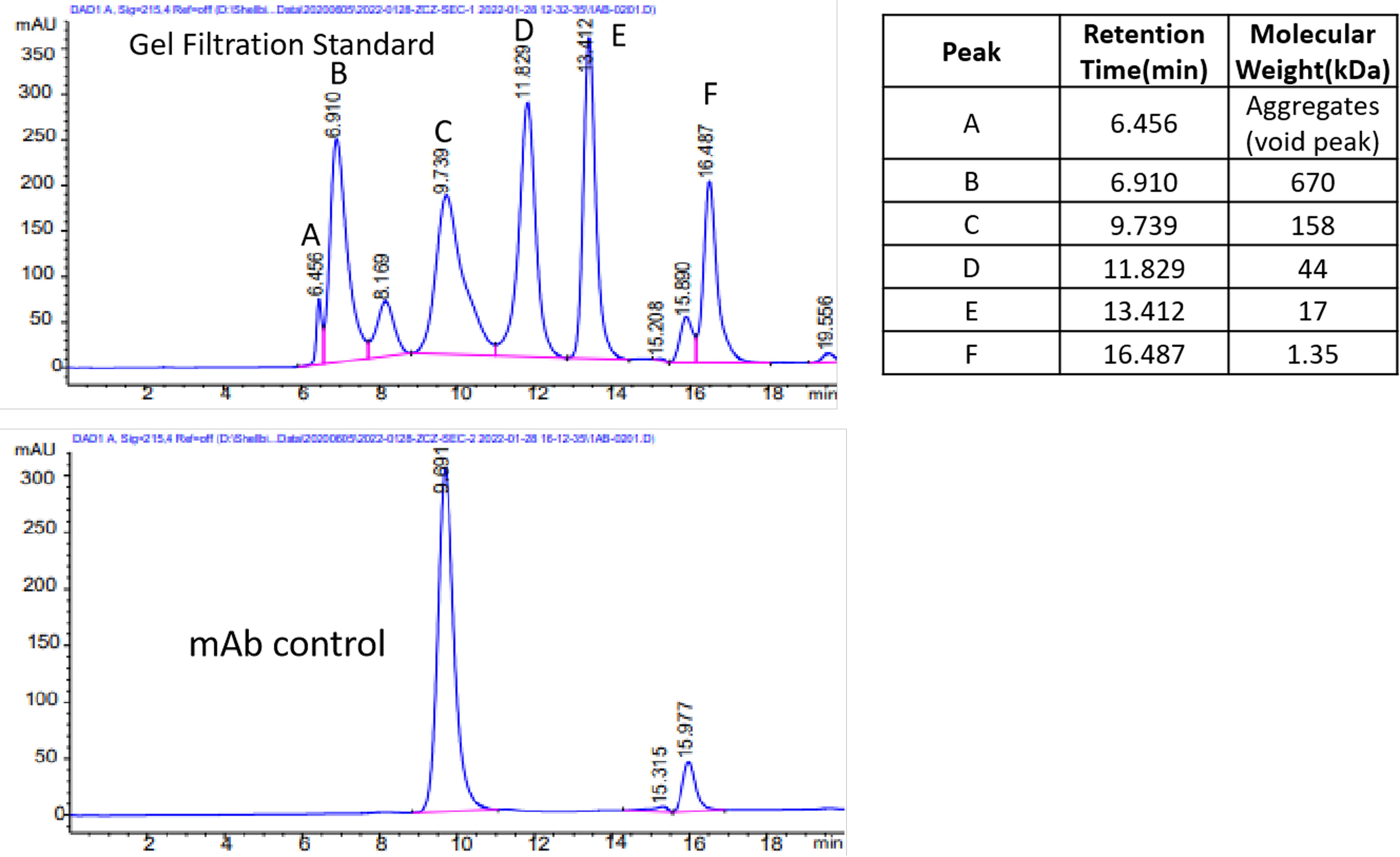
**

**Supplement Figure 3. SEC-HPLC standard.**


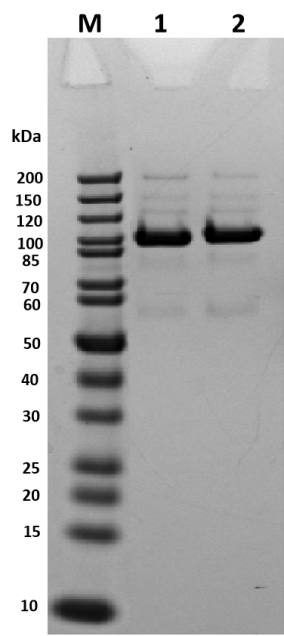


**Supplement Figure 4. Purification of an antibody with a non-native disulfide bond.** SDS-PAGE of purified antibodies by protein A chromatography. Lane 1 is the antibody obtained using the design without non-native disulfide bond. Lane 2 is the second antibody that has additional mutations and a non-native disulfide bond.

**Supplement Table 1. EC50 and the maximum response (Top) of PD-1xLAG3 TiMab and the corresponding parental antibodies.**

|  | PD-1 | | LAG3 | |
| --- | --- | --- | --- | --- |
|  | EC50 (nM) | Top | EC50 (nM) | Top |
| TiMab | 2.756 | 4501 | 3.366 | 4252 |
| PD-1 | 2.545 | 4704 |  |  |
| LAG3 |  |  | 3.086 | 4232 |


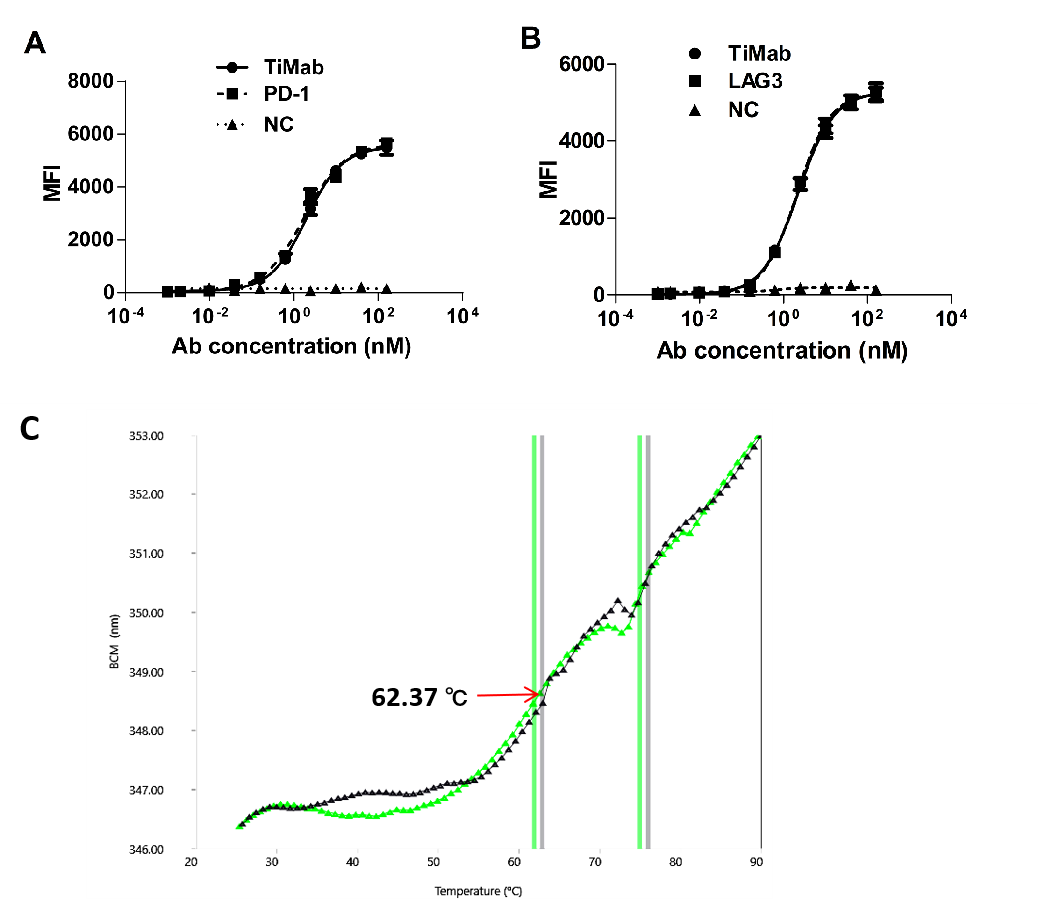


**Supplement Figure 5. Thermostability analysis of PD-1xLAG3 TiMab.** A and B, binding results with PD-1 and LAG3 after 21-day treatment at 40°C. There was no significant change in the antibody binding ability after treatment. C, The Tm1 of protein was quantitatively detected by Uncle biologics stability screening platform. The red arrow indicates an average Tm1 value of 62.37°C.

**Supplement Table 2. Thermostability of PD-1xLAG3 TiMab at 40ºC. The concentration and purity of the antibodies at different time points were measured, respectively. Conc., concentration**

|  |  | IgG4 S228P isotype | PD-1xLAG-3 TiMab |
| --- | --- | --- | --- |
| Conc. (mg/mL) | Day 0 | 1.12 | 1.11 |
|  | Day 1 | 1.13 | 1.09 |
|  | Day 7 | 1.15 | 1.12 |
|  | Day 14 | 1.17 | 1.15 |
|  | Day 21 | 1.19 | 1.10 |
| SEC-HPLC | Day 0 | 98.18% | 95.65% |
|  | Day 1 | 97.40% | 94.48% |
|  | Day 7 | 96.87% | 93.67% |
|  | Day 14 | 96.36% | 92.85% |
|  | Day 21 | 95.34% | 92.19% |


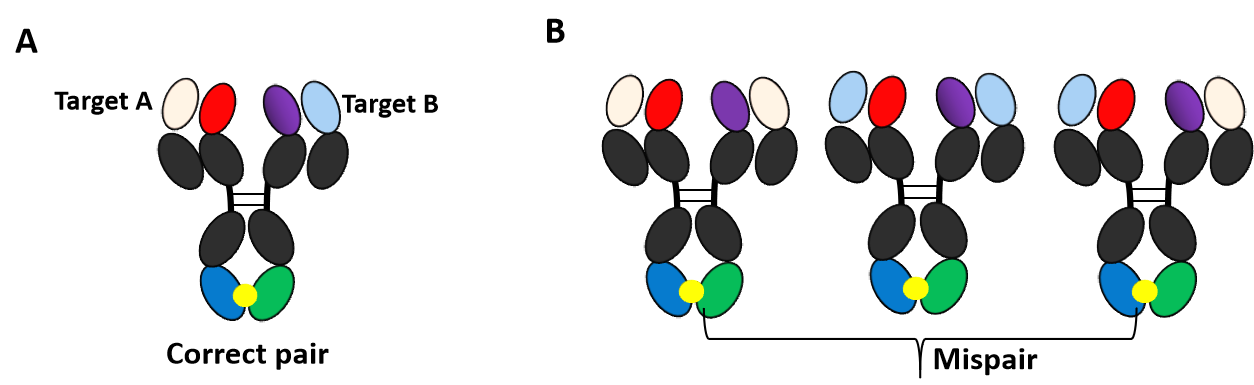


**Supplement Figure 6. Free combination of light and heavy chains in single cell transfection.** A, The form of correct pair. B, Possible forms of mispair


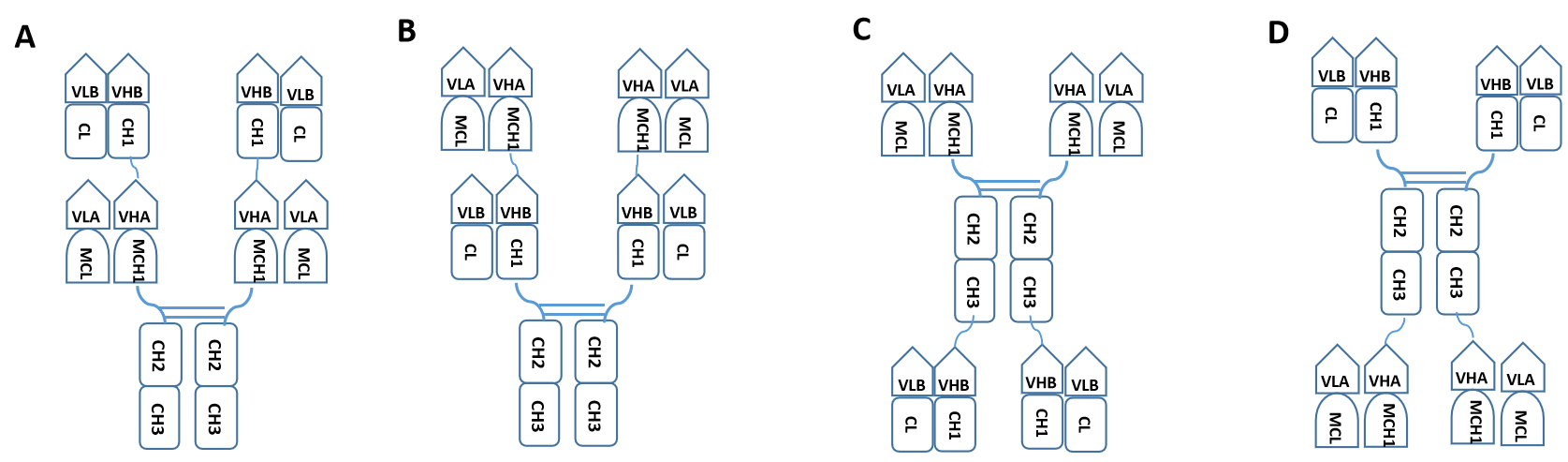


**Supplement Figure 7. Schematic diagram of 4 tetrevalent (2+2) IgG-like bispecific antibodies**. A, Adding Fab to N-terminal. B, Adding chimeric Fab to N-terminal. C, Adding Fab to C-terminal. D, Adding chimeric Fab to C-terminal.


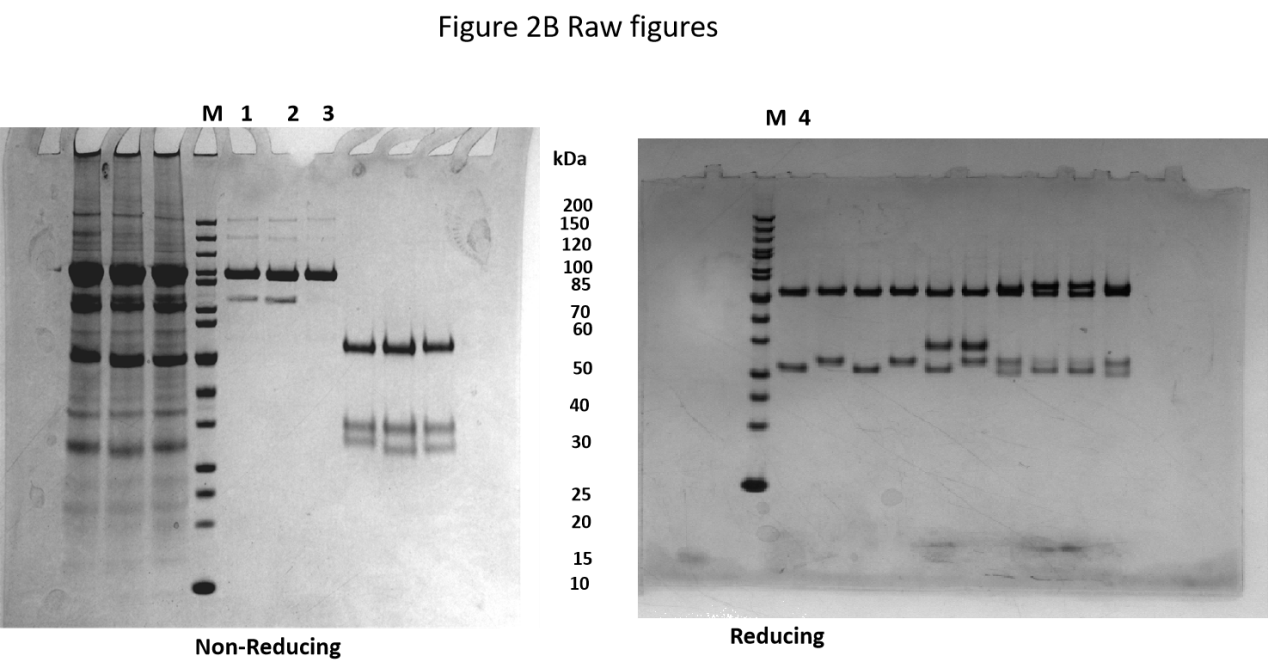


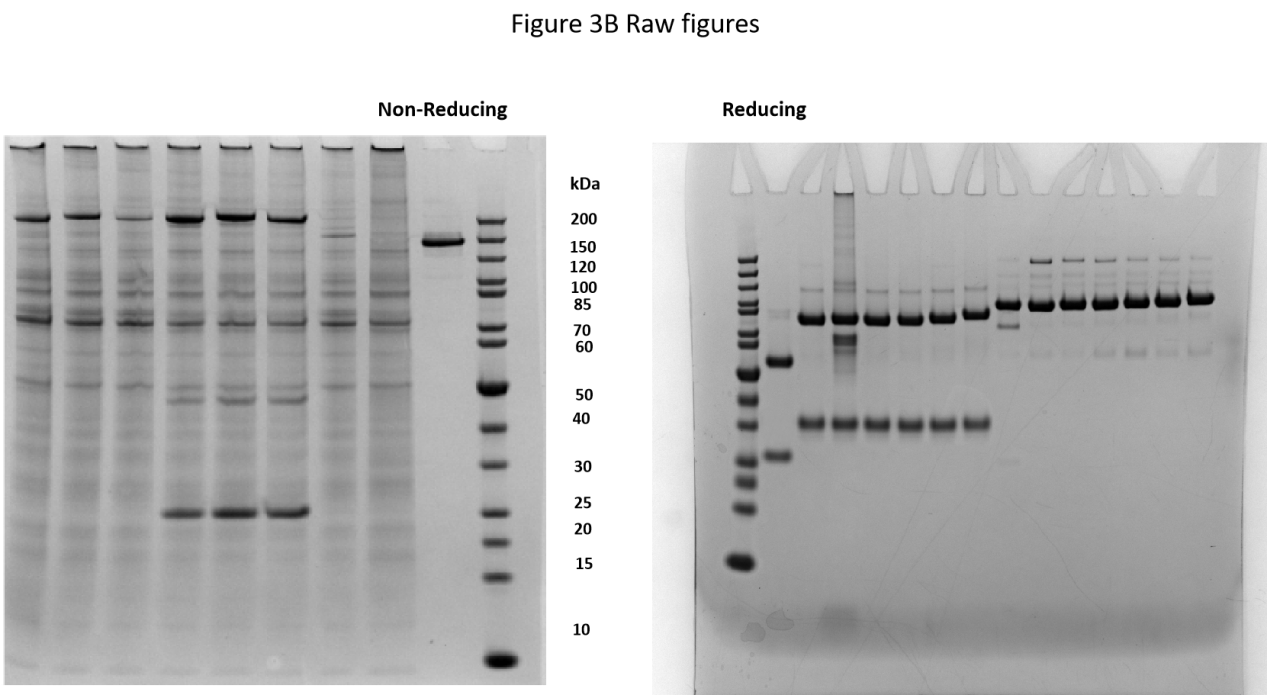


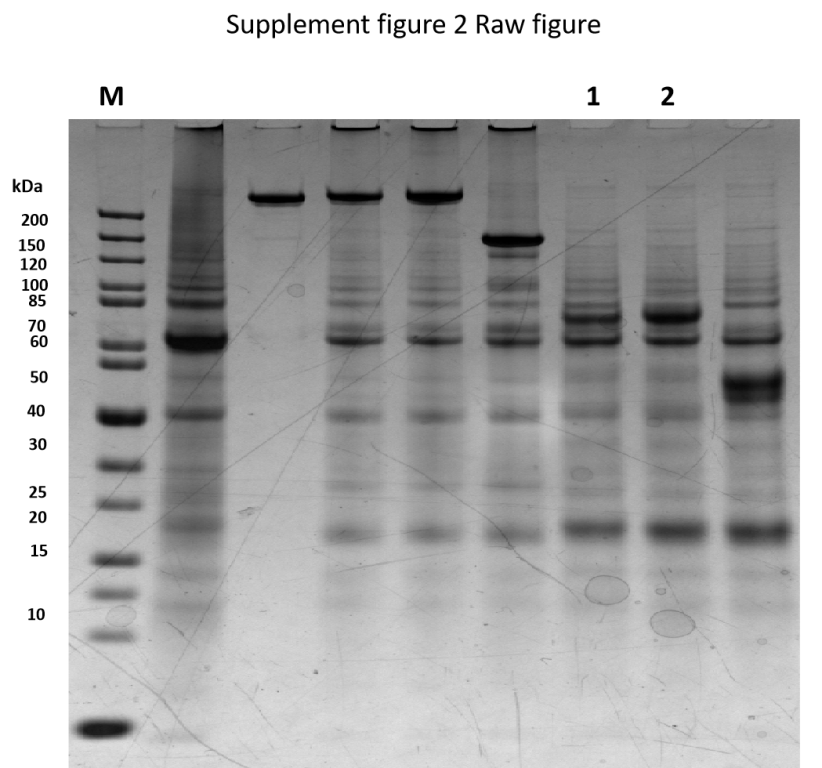


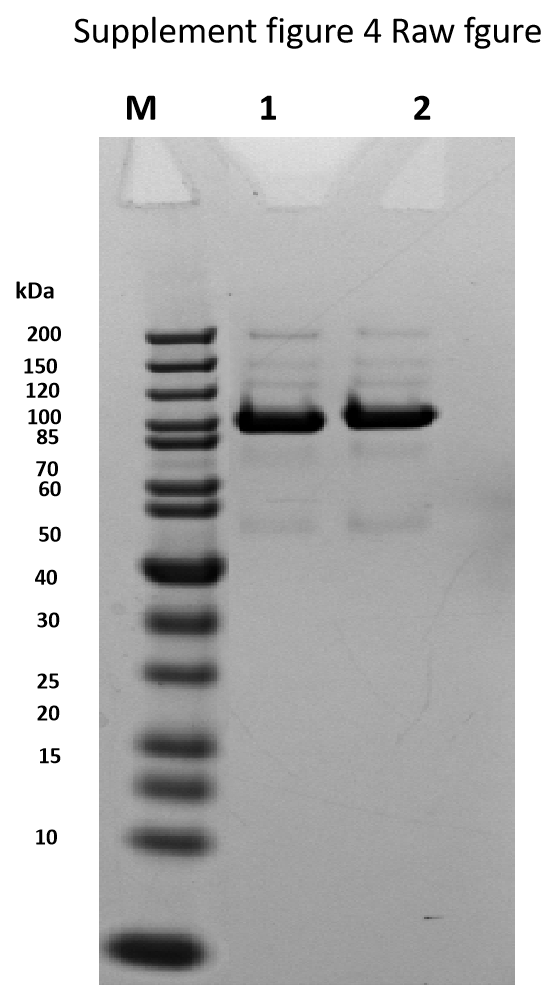


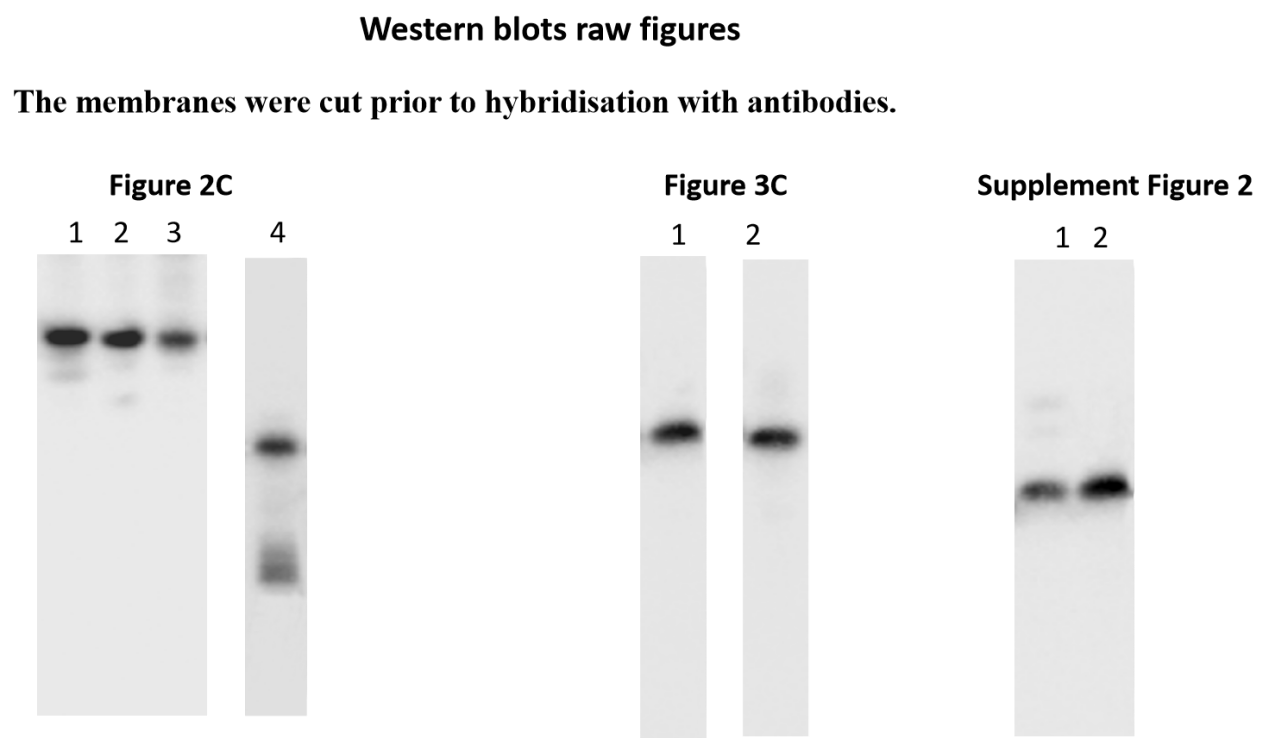

Supplement: Supplementary file 1 — Supplementary Information. [file 41598_2021_97393_MOESM1_ESM.docx]
